# Structural and molecular dynamics insights into the competitive inhibition of the platelet-activating factor receptor by acyl-PAF

**DOI:** 10.1016/j.jbc.2025.110831

**Published:** 2025-10-16

**Authors:** Shao-Chi Hung, Chih-Chieh Chen, Hua-Chen Chan, Mei-Lin Chan, You Shan Ngui, Jia-Bin Mei, Yi-Wen Huang, Vyala Hanumanthareddy Chaithra, Gopal Kedihithlu Marathe, Liang-Yin Ke

**Affiliations:** 1 Department of Medical Laboratory Science and Biotechnology, College of Health Sciences, Kaohsiung Medical University, Kaohsiung, Taiwan; 2 Institute of Medical Science and Technology, National Sun Yat-sen University, Kaohsiung, Taiwan; 3 Department of Medical Laboratory Science, College of Medicine, I-Shou University, Kaohsiung, Taiwan; 4 Department of Surgery, MacKay Memorial Hospital, Taipei, Taiwan; 5 Department of Medicine, MacKay Medical College, New Taipei City, Taiwan; 6 Department of Medical Research, Mackay Memorial Hospital, New Taipei City, Taiwan; 7 Department of Studies in Biochemistry and Molecular Biology, University of Mysore, Manasagangothri, Mysuru, India; 8 Graduate Institute of Medicine, College of Medicine & Drug Development and Value Creation Research Center, Kaohsiung Medical University, Kaohsiung, Taiwan; 9 Department of Laboratory Medicine, Kaohsiung Medical University Hospital, Kaohsiung Medical University, Kaohsiung, Taiwan

**Keywords:** platelet-activating factor (PAF), PAF receptor (PAFR), acyl-PAF, molecular dynamic simulation

## Abstract

Platelet-activating factor (PAF) promotes cellular activation, inflammation, and platelet aggregation, leading to acute thrombosis. The remodeling pathway swiftly produces PAF upon activation, establishing intracellular signaling *via* the PAF receptor (PAFR). Alkyl-PAF exhibits the aforementioned inflammatory properties; conversely, its structural analog acyl-PAF elicits a diminished response and functions as an endogenous PAFR antagonist. The underlying mechanism remains undefined. We conducted the molecular dynamics simulation to elucidate the interactions of acyl-PAF or alkyl-PAF on PAFR. Results showed that alkyl-PAF and acyl-PAF share the same binding pocket. However, acyl-PAF increases the radius of gyration and root-mean-square deviation of PAFR, resulting in structural instability. Root-mean-square fluctuation and solvent-accessible surface area analysis indicated that acyl-PAF reduces flexibility at the PAFR-G protein interface and interaction with key residues involved in signaling, suggesting impaired G protein dissociation and downstream transduction. Acyl-PAF forms stronger interaction with PAFR by increasing hydrogen bonds and favorable Gibbs free energy (*ΔG*) changes. The Markov state model analysis confirmed these findings, showing that acyl-PAF attaches to PAFR with a lower equilibrium dissociation constant, greater association rate, and lower dissociation rate. In conclusion, acyl-PAF induces structural instability and diminishes signaling effectiveness despite a high binding affinity. This study dissects the intricate interactions within PAFR signaling pathway and offers new insights into potential therapeutic interventions.

Platelet-activating factor (PAF; 1-*O*-alkyl-2-acetyl-*sn*-glyceryl-3-phosphorylcholine, also known as alkyl-PAF) is a pro-inflammatory bioactive lipid that activates endothelial cells, immune cells, and platelets (1, 2, 3). It may provoke platelet aggregation, clot formation, and inflammation, leading to acute thrombotic disorders (4, 5). Besides, it is involved in various pathological responses, including asthma, anaphylaxis, and cancers (6, 7, 8, 9). Alkyl-PAF is synthesized predominantly through the *de novo* pathway from precursor 1-alkyl-2-lyso-*sn*-glycero-3-phosphate, which contributes to basal levels of PAF (10). In response to stimulation, alkyl-PAF is swiftly produced intracellularly through the remodeling pathway, utilizing membrane phospholipid as the primary precursor (11, 12). Upon release, it interacts with neighboring cells through the PAF receptor (PAFR), a G protein-coupled receptor (GPCR) coupled with the G(i) and G(q) subtypes of heterotrimeric G proteins (13, 14). The binding of PAF and PAFR triggers a cascade of intracellular signaling, encompassing phospholipase C (PLC) activation, calcium mobilization, and NFκB/MAPK (nuclear factor-kappa B/mitogen-activated protein kinases) signaling, which ultimately results in vascular permeability alteration, leukocyte adhesion, and endothelial dysfunction (15). In circulation, PAF is highly enriched in the electronegative low-density lipoprotein (LDL(−)) particles. It augments the expression of CX3CR1 and CD16 in human monocytes, promotes endothelial activation, and contributes to atherosclerosis and acute myocardial infarction (5, 16).

Acyl-PAF (1-*O*-acyl-2-acetyl-*sn*-glyceryl-3-phosphorylcholine) is a structural analog of PAF that contains an ester-linked acyl chain at the *sn*-1 position, unlike the ether-linked alkyl chain present in canonical PAF (alkyl-PAF) (17, 18). Acyl-PAF is simultaneously produced alongside alkyl-PAF through the remodeling pathway, using phosphatidylcholine (PC) as a precursor, and is predominantly synthesized by endothelial cells (19). The subtle structural variation dramatically diminishes biological activity, with acyl-PAF exhibiting nearly 100-fold to 2000-fold lower pro-inflammatory potency than alkyl-PAF (20, 21). Interestingly, acyl-PAF exerts its biological effects by interacting with the same receptor, the PAFR. Acyl-PAF has been demonstrated to counteract alkyl-PAF-induced acute mortality in Swiss albino mice (22). Moreover, co-treatment with only trace amounts of acyl-PAF is sufficient to elicit anti-inflammatory effects and to attenuate alkyl-PAF-induced platelet aggregation, suggesting its potential role as a partial antagonist and competitive relationship with alkyl-PAF (22). However, the molecular basis of its antagonistic mechanism and the dynamic interplay among alkyl-PAF, acyl-PAF, and PAFR are yet inadequately comprehended.

To tackle this unmet clinical issue, we aimed to utilize Graphics Processing Unit (GPU)-accelerated computational molecular dynamics (MD) simulation software to investigate the binding interactions of PAF analogs with the PAFR. MD simulation tracks the movement of individual atoms on solvated biomolecular systems, potentially offering femtosecond-resolved insights into conformational fluctuations, transient ligand-receptor contacts, and underlying energetic landscapes that are predominantly unattainable by experimental techniques (23). Notably, GROMACS is particularly well-suited for simulating proteins, lipids, and nucleic acids (24). Therefore, we used it to analyze protein structural dynamics, ligand–residue interactions, and binding affinities to elucidate the mechanistic basis underlying the functional divergence between alkyl-PAF and acyl-PAF. Furthermore, we explored the clinical implications of blocking PAF signaling in inflammatory diseases. Our findings may offer valuable insights for the development of innovative anti-inflammatory molecules with therapeutic potential.

## Results

### Competitive binding of alkyl-PAF and acyl-PAF to the same pocket of PAFR

**Figure 1:**
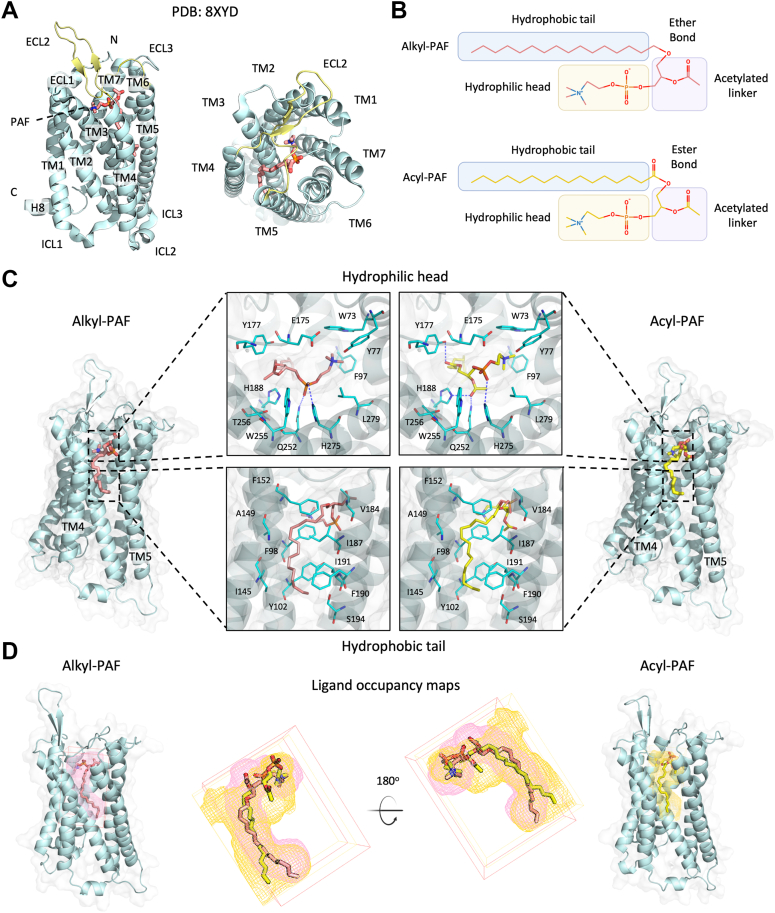
**Molecular docking and binding interactions of acyl-PAF and alkyl-PAF with PAFR**. *A*, the side view of the PAFR structure (PDB: 8XYD, Chain *A*, *cyan*) with C16 PAF (*red*) consists of seven transmembrane helices (TM1-TM7), one short helix (H8), 3 extracellular loops (ECL1-ECL3), and 3 intracellular loops (ICL1-ICL3). The extracellular (*top-down*) view of the PAFR structure, highlighting the ECL2 in *yellow*. *B*, the chemical structure of alkyl-PAF C-16 (ether bond) and acyl-PAF C-16 (ester bond). Both molecules share a hydrophobic tail, an acetylated linker, and a hydrophilic head. *C*, docking pose and binding interactions of alkyl-PAF (*pink*) and acyl-PAF (*yellow*) with PAFR (*pale cyan*). Oxygen atoms are shown in *red*, nitrogen in *blue*, and phosphorus in *orange*. Key interacting residues in the binding pocket are highlighted as *cyan* sticks. The *blue dashed lines* indicate hydrogen bond interactions. *D*, time-averaged ligand occupancy density maps showing the spatial distribution of alkyl-PAF (*pink mesh*) and acyl-PAF (*yellow mesh*) within the receptor binding pocket. The PAFR is displayed in cyan cartoon, with alkyl-PAF (*red sticks*) or acyl-PAF (*yellow sticks*). PAF, platelet-activating factor; PAFR, platelet-activating factor receptor.

The 3D structure of human PAFR (PDB: 8XYD, Chain A) bound to platelet-activating factor was obtained from the Protein Data Bank and visualized using PyMOL (25). It comprises seven transmembrane α-helices (TM1-TM7), a short intracellular helix (H8), 3 extracellular loops (ECL1-ECL3), and 3 intracellular loops (ICL1-ICL3), featuring an extracellular N-terminus and an intracellular C-terminus (Fig. 1*A*, left panel). An extracellular top view of PAFR reveals a central binding cavity encircled by transmembrane helices (TM1-TM7), with the ECL2 structure forming the primary ligand entry site (Fig. 1*A*, right panel). C16 alkyl-PAF and acyl-PAF are phospholipids with a hydrophobic carbon tail, an acetylated glycerol backbone, and a hydrophilic phosphorylcholine head group (Fig. 1*B*). The distinction lies at the *sn*-1 position, characterized by an ether bond in alkyl-PAF or an ester linkage in acyl-PAF (26). The 3D chemical structures of C16 alkyl-PAF and acyl-PAF were simultaneously generated for subsequent study.

We applied computational molecular docking for pose searching to compare the binding of alkyl-PAF and acyl-PAF on PAFR. Results showed that both PAF ligands occupied the orthosteric binding pocket, with their hydrophobic tails extending into the cleft between transmembrane helices TM4 and TM5. The amino acids within a 4 Å radius were designated (Fig. 1*C*). For alkyl-PAF binding with PAFR, the positively charged choline moiety of the polar head was oriented towards helix TM2 in proximity to residues W73 (TM2), Y77 (ECL1), F97 (TM3), and E175 (ECL2) of PAFR (Fig. 1*C*, upper left panel, hydrophilic head). The negatively charged phosphate group formed polar interactions with Q252 (TM6) and H275 (ECL3) of PAFR, aiding in the stability of the head group (Fig. 1*C*, upper left panel, dashed line). In parallel, acyl-PAF exhibited a comparable conformation, with the choline moiety of the head group and carbon tail positioned similarly to alkyl-PAF (Fig. 1*C*, upper right panel). Nonetheless, the chemical distinction of acyl-PAF permitted the acyl group, acetylated linker, and hydrophilic head to interact uniquely with various amino acids and face different orientations. The phosphate group of acyl-PAF exclusively established a hydrogen bond with H275 (ECL3) because of its displacement from helix TM6, whereas the acetylated linker developed hydrogen connections with H188 (TM5) and Q252 (TM6). Furthermore, the ester linkage, featuring carbonyl oxygen, created an extra hydrogen bond with Y177 (ECL2) (Fig. 1*C*, upper right panel). Despite acyl-PAF establishing more hydrogen bonds with PAFR, both PAF ligands adopted a similar binding orientation within the orthosteric pocket of PAFR, providing structural support for their competitive binding. To further define the binding region, we computed ligand occupancy maps from the MD trajectories. The resulting isomeshes showed a high degree of spatial overlap between alkyl-PAF and acyl-PAF, consistent with a shared pocket (Fig. 1*D*).

### Altered structural dynamics of PAFR upon acyl-PAF binding

**Figure 2:**
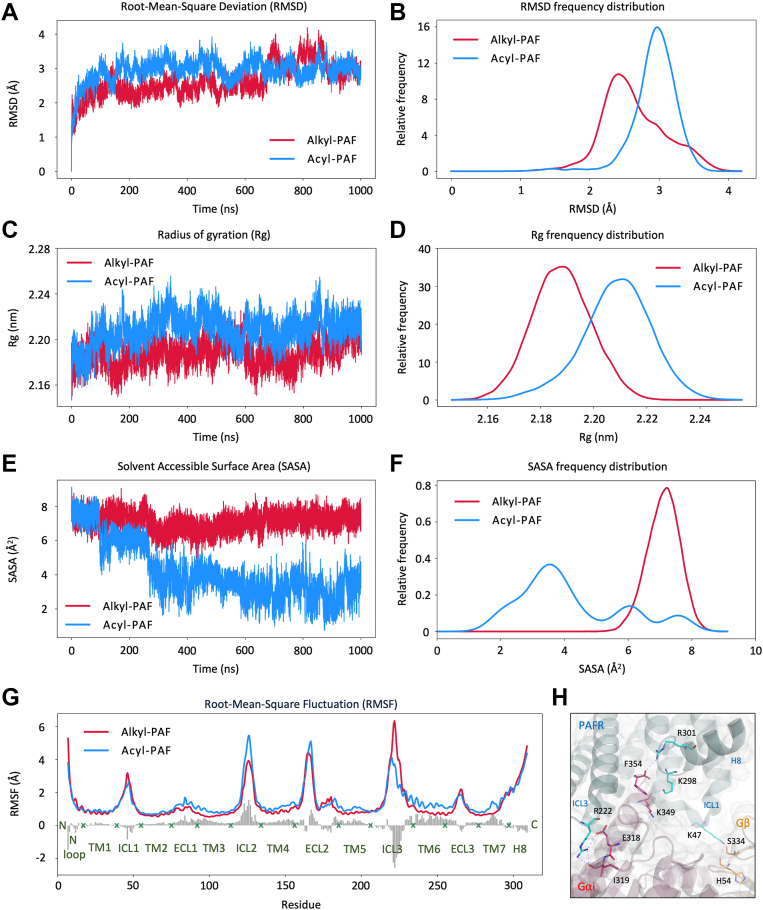
**Comparative analysis of PAFR structural dynamics and key residue interactions in alkyl-PAF and acyl-PAF systems**. *A*, time evolution and (*B*) frequency distribution of the PAFR backbone RMSD. *C*, time evolution and (*D*) frequency distribution of the PAFR backbone Rg. *E*, time evolution and (*F*) frequency distribution of the SASA between PAF ligands (acyl-PAF and alkyl-PAF) and key PAFR residues (W73, F97, F174, E175, W255, and H275). *G*, residue-level RMSF profile of PAFR. *Gray bars* represent fluctuation differences between residues in the two ligand-bound systems (acyl-PAF minus alkyl-PAF); positive values indicate higher fluctuations in the acyl-PAF system. *H*, structural representation of the PAFR-G protein interface. Residues in PAFR (R222, K298, and R301) are shown in *cyan*; residues in the Gα subunit (E318, I319, F354, and K349) in *red*; and residues in the Gβ subunit (H54 and S334) in *orange*. *Blue dashed lines* indicate hydrogen bond interactions. PAF, platelet-activating factor; PAFR, platelet-activating factor receptor; Rg, radius of gyration; RMSD, root-mean-square deviation; RMSF, root-mean-square deviation fluctuation; SASA, solvent-accessible surface area.

PAFR belongs to the GPCR superfamily, whose conformational flexibility is essential for effective G protein coupling and downstream signaling (27, 28). We conducted independent 1000 ns computational MD simulations to dissect PAF-induced structural alterations in alkyl-PAF and acyl-PAF-bound PAFR complexes. The acyl-PAF binding resulted in a notable increase in the structural fluctuation of PAFR, as reflected by higher PAFR backbone root-mean-square deviation (RMSD, 2.94 ± 0.32 Å *versus* 2.64 ± 0.45 Å) (Fig. 2, *A* and *B*) and radius of gyration (Rg, 2.21 ± 0.01 nm *versus* 2.19 ± 0.01 nm) (Fig. 2, *C* and *D*) compared to alkyl-PAF, indicating overall structural destabilization. To further probe ligand-receptor interface dynamics, we calculated the solvent-accessible surface area (SASA) between the PAFs and six key PAFR residues (W73, F97, F174, E175, W255, and H275), which have been previously recognized as essential for PAFR activation (25). After reaching equilibrium (∼200 ns), acyl-PAF displayed a markedly reduced contact area (4.19 ± 1.64 Å^2^) compared to alkyl-PAF (7.11 ± 0.51 Å^2^) (Fig. 2, *E* and *F*), suggesting impaired engagement with functional amino acids.

Residue-level fluctuations were analyzed using root-mean-square fluctuation (RMSF) profiles. Despite acyl-PAF increasing overall structural fluctuations in PAFR (1.45 ± 0.89 Å *versus* 1.32 ± 1.01 Å), it paradoxically led to decreased RMSF values in regions critical for G protein interaction, specifically ICL1, ICL3, and H8 (Fig. 2*G*, Gray bars indicate RMSF_acyl-PAF_ – RMSF_alkyl-PAF_). To elucidate the role of ICL1, ICL3, and H8 of PAFR in interaction with the G protein, the hydrogen bonds between the two molecules were contextualized in Figure 2*H*. For example, the R301 (H8) of PAFR (shown in cyan) formed a hydrogen bond with F354 (Gαi) of the Gα subunit (shown in red). Similarly, K298 (H8) - K349 (Gαi), R222 (ICL3) - E318/I319 (Gαi), and K47 (ICL1) - S334/H54 (Gβ, shown in orange) represented PAFR–G protein interactions.

**Figure 3:**
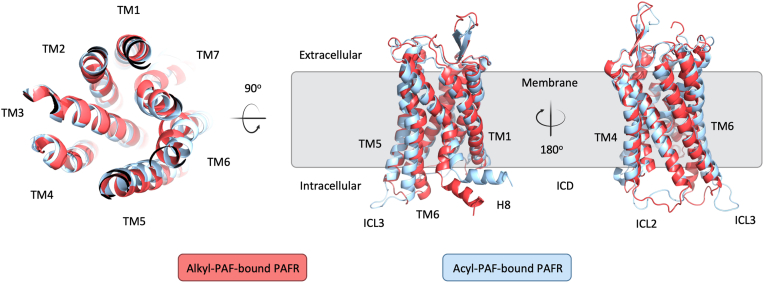
**Structure alignment of the alkyl-PAF-bound PAFR with acyl-PAF-bound PAFR**. Representative conformations of alkyl-PAF-bound (*red*) and acyl-PAF-bound (*blue*) PAFR are shown from three different viewpoints: extracellular top-down (*left panel*), side view perpendicular to the membrane (*center*), and rotated 180° around the vertical axis (*right panel*). The *gray box* indicates the approximate location of the membrane bilayer. Conformational differences in the intracellular domain (ICD), particularly in the TM6, ICL2, ICL3, and H8 regions. ICD, intracellular domain.

To visualize the conformational alternation, we aligned the PAFR structure after its binding with alkyl-PAF or acyl-PAF. The results indicated that, while the top and side views of the extracellular domain exhibited no significant differences, the intracellular structures were markedly diverse (Fig. 3). Specifically, TM6 was displaced outward to different extents, and H8 was reoriented to different angles between the alkyl-PAF and acyl-PAF-bound structures. The conformations of intracellular loops ICL2 and ICL3 were also distinct. Collectively, these structural insights suggest that the alkyl-PAF and acyl-PAF stabilize different intracellular states of PAFR and may lead to different downstream signaling outcomes.

### Higher binding affinity of acyl-PAF to PAFR

**Figure 4:**
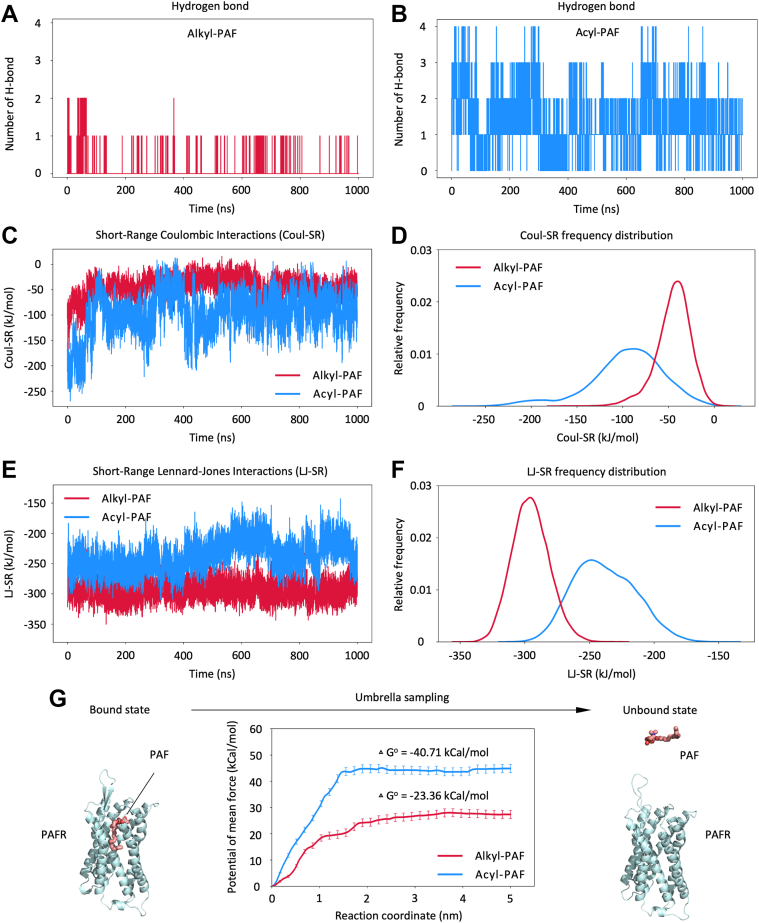
**Comparative analysis of binding affinity and interaction energies between alkyl-PAF and acyl-PAF**. *A*, the number of hydrogen bonds between PAFR and alkyl-PAF or (*B*) acyl-PAF. *C*, time evolution and (*D*) frequency distribution of the Coul-SR between PAFR and PAF ligands (acyl-PAF and alkyl-PAF). *E*, time evolution and (*F*) frequency distribution of the LJ-SR. *G*, average grid PMF based on umbrella sampling simulation with absolute binding free energy (Δ*G*°) (bound to unbound). The horizontal axis represents the pulling distance of the ligand from its initial bound position. The PAF molecule is shown as a *red stick*, and the PAFR in *pale cyan*. Coul-SR, short-range coulombic interactions; LJ-SR, short-range Lennard-Jones interactions; PAF, platelet-activating factor; PAFR, platelet-activating factor receptor; PMF, potential of mean force.

PAF-PAFR signaling is extensively associated with inflammatory responses (29); hence, synthetic small-molecule antagonists such as Rupatadine and Lexipafant are known for competitively binding to PAFR with high affinity (15). To assess whether acyl-PAF may function as an endogenous antagonist with anti-inflammatory properties, we analyzed its binding interactions with PAFR, focusing on hydrogen bonding, interaction energy, and free energy calculations in comparison to alkyl-PAF. Hydrogen bond analysis revealed that acyl-PAF exhibited a significantly higher propensity to form hydrogen bonds with PAFR (1.30 ± 0.82) than alkyl-PAF (0.07 ± 0.28) (Fig. 4, *A* and *B*). This finding aligned with the endpoint docking results (Fig. 1*D*) and may be attributed to the presence of extra oxygen atoms in the acyl moiety, which acted as possible hydrogen bond acceptors. The augmented hydrogen bonding pattern was further evidenced by the stronger short-range Coulombic interactions (Coul-SR) observed for acyl-PAF (−95.82 ± 41.03 kJ/mol) compared to alkyl-PAF (−44.79 ± 19.12 kJ/mol) (Fig. 4, *C* and *D*). In contrast, the short-range Lennard-Jones interactions (LJ-SR), primarily representing van der Waals (vdW) and hydrophobic contacts, exhibited reduced strength for acyl-PAF (−238.48 ± 23.93 kJ/mol) compared to alkyl-PAF (−295.47 ± 14.26 kJ/mol), suggesting a decrease in hydrophobic contributions (Fig. 4, *E* and *F*). Nonetheless, umbrella sampling revealed a more favorable absolute binding free energy for acyl-PAF compared to alkyl-PAF (−23.36 kCal/mol vs. −40.71 kCal/mol; bound to unbound), indicating a stronger overall affinity for PAFR (Fig. 4*G*). These findings support the hypothesis that acyl-PAF may function as a partial endogenous antagonist with anti-inflammatory potential through its strong yet low productive binding.

### Rapid binding and prolonged residence characterize acyl-PAF-PAFR interaction

**Figure 5:**
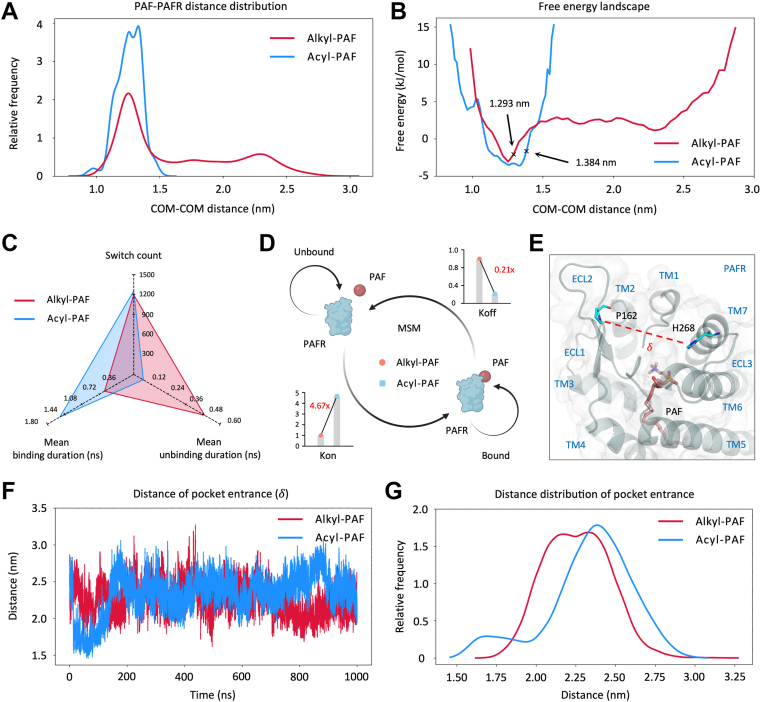
**Comparison of alkyl-PAF and acyl-PAF binding kinetics and pocket interactions with PAFR**. *A*, relative frequency distribution of the distance between PAF ligands (alkyl-PAF and acyl-PAF) and PAFR. *B,* Free energy landscape (FEL) of alkyl-PAF and acyl-PAF systems based on PAF-PAFR distance. The *arrow* indicates the threshold used to define the bound and unbound states. *C*, the radar chart compares binding behaviors, including switch count, mean binding duration, and mean unbinding duration. *D*, the schematic diagram illustrates state transitions and calculated kinetic parameters, including Kon and Koff, derived from the Markov state model. Alkyl-PAF is shown in *red* and acyl-PAF in *skyblue*. *E*, structural representation of the PAFR binding pocket entrance. *δ* denotes the distance between P162 (ECL2) and H268 (ECL3). *F*, time evolution and (*G*) frequency distribution of the pocket distance (*δ*). Kd, equilibrium dissociation constant; Koff, dissociation rate constant; Kon, association rate constant; PAF, platelet-activating factor; PAFR, platelet-activating factor receptor.

Alongside the thermodynamic stability of the bound complex, we assessed the kinetic parameters, including association rate (Kon), dissociation rate (Koff), and ligand residence time (RT), to evaluate the ligand effectiveness of acyl-PAF and alkyl-PAF on PAFR (30). The distance distribution profile indicated that acyl-PAF mostly resided at the PAFR binding site (1.26 ± 0.10 nm), signifying a stable association. In contrast, alkyl-PAF displayed a broader distance distribution (1.62 ± 0.46 nm) with a distal population indicative of unbound states, suggesting a higher frequency of dissociation occurrences (Fig. 5*A*). The thresholds distinguishing bound and unbound states were determined using the second-derivative analysis of the free energy landscape, with cutoff values of 1.384 nm for acyl-PAF and 1.293 nm for alkyl-PAF (Fig. 5*B*). A higher threshold signified an elevated energy barrier, hindering transitions between bound and unbound states and favoring stability in one conformation. To depict the dynamic transitions of the two ligands on PAFR, a radar plot was generated (Fig. 5*C*). In the MSM analysis, both ligands exhibited a comparable number of switching events (1196 *versus* 1255); however, acyl-PAF showed a longer mean binding duration (1.54 ± 11.89 ns *versus* 0.61 ± 3.22 ns) and a shorter mean unbinding duration (0.13 ± 0.59 ns *versus* 0.98 ± 12.34 ns) compared to alkyl-PAF. According to the transition probability matrix obtained from the MSM model, acyl-PAF had a 4.67-fold greater Kon and a 0.21-fold reduced Koff in comparison to alkyl-PAF (Fig. 5*D*). These results suggest that acyl-PAF binds more rapidly and exhibits a prolonged residence time at the PAFR than alkyl-PAF. To further assess the accessibility of the orthosteric site, we evaluated the entry width (*δ*) of the PAFR binding pocket, defined by the distance between residue P162 (ECL2) and H268 (ECL3) (Fig. 5*F*). Trajectory analysis revealed that acyl-PAF formed an expanded pocket entry compared to alkyl-PAF (2.34 ± 0.28 nm *versus* 2.26 ± 0.21 nm), implying a more accessible binding pathway (Fig.5, *F* and *G*). Based on the bound-unbound threshold defined by the free energy landscape, each system was separated into bound and unbound ensembles. In the alkyl-PAF system, the bound state (median = 2.24 nm) displayed a smaller pocket opening compared with the unbound state (median = 2.27 nm), explaining the bimodal distribution observed in the full ensemble (Fig. S1*A*). In contrast, the acyl-PAF system (unbound, median = 2.35 nm; bound, median = 2.38 nm) predominantly sampled the bound state due to its higher binding affinity, resulting in an overall distribution similar to the bound ensemble (Fig. S1*B*). These findings underscore the high binding affinity of acyl-PAF to PAFR, characterized by rapid association and limited dissociation, hence reinforcing its role as an endogenous antagonist.

## Discussion

This study elucidates the structural and dynamic perspective on the contrasting roles of acyl-PAF and alkyl-PAF in modulating PAFR signaling, which can clarify the contradictory effects of acyl-PAF on PAFR signaling documented in previous studies (2, 22). Through computational molecular docking, we noted that acyl-PAF and alkyl-PAF occupied a nearly identical binding pose on PAFR. Their hydrophilic headgroups were positioned at the entrance of the orthosteric pocket, whereas carbon tails stretched between transmembrane helices TM4 and TM5 (Fig. 1*C*). Ligand occupancy analysis further validated a high degree of spatial overlap between the alkyl-PAF and the acyl-PAF (Fig. 1*D*). This structural and spatial similarity highlights the competitive relationship between the two PAF analogs. Although acyl-PAF exhibited higher affinity, it destabilized the PAFR, hence hampering downstream signaling (Figure 2, Figure 3, Figure 4). Besides, Markov state model (MSM) indicated that acyl-PAF preferentially occupied the orthosteric binding site of PAFR and exhibited a reduced dissociation rate, underscoring its kinetic stability. Collectively, these findings suggest that acyl-PAF may serve as an endogenous antagonist, exerting anti-inflammatory effects by enhancing receptor binding without facilitating productive activation. These findings aligned with prior *in vivo* research revealing that the Swiss albino mice pre-treated with acyl-PAF might salvage a lethal dose of alkyl-PAF (22). Furthermore, similar patterns of competitive binding have been shown in other GPCR systems. Agonists and antagonists that target the β2-adrenergic receptor (β2AR) and the cannabinoid receptor 1 (CB1) bind to virtually similar areas within the orthosteric site while eliciting contrary functional effects (31, 32). The results reinforce that ligands occupying the same binding site can stabilize distinct receptor conformations, ultimately leading to divergent signaling responses.

Although it is well known that PAFR facilitates inflammatory responses by transmitting extracellular signals into intracellular G protein (33), this work demonstrated that the binding of acyl-PAF diminished downstream signaling by destabilizing the PAFR structure, reducing its interactions with functional residues, and causing localized rigidification at the G protein interface, primarily within the ICL3 motif (Fig. 2). Further structural comparisons revealed that alkyl-PAF and acyl-PAF stabilized distinct receptor conformations, especially at the intracellular domain (ICD), with notable differences in the orientations of TM6, H8, and intracellular loops (Fig. 3). Given that conformational rearrangement of the ICD is crucial for G protein engagement and subunit dissociation (34, 35), these variations likely cause the two ligands to have different signaling outcomes. This perspective has been affirmed by an additional MD simulation with WEB2086 (Apafant), a potent PAFR antagonist, which also elicited structurally unstable (Fig. S2) and stiffening at the G protein-GPCR coupling interface, further implicating ICD rigidification as a common mechanism for signal suppression (Fig. S3).

Apart from structural alterations, energy decomposition analysis revealed that acyl-PAF exhibited stronger electrostatic interactions but significantly weaker van der Waals contributions than alkyl-PAF (Fig. 4, *C*–*F*), suggesting a less favorable fit within the hydrophobic binding pocket. Consistently, pocket entrance analysis showed that binding of alkyl-PAF was associated with a smaller pocket opening, reflecting tighter accommodation of the shorter alkyl group, whereas acyl-PAF binding required expansion of the entrance to accommodate its acyl linkage (Fig. S1). These results aligned with the noted decrease in contact surface area (Fig. 2, *E* and *F*) and the attenuation of downstream signaling. Furthermore, acyl-PAF bound at a somewhat greater distance from the receptor core (1.34 nm compared to 1.25 nm), which presumably undermined hydrophobic interactions and is essential for productive receptor activation (Fig. 5*B*). Notably, owing to its distinct orientation and ester moiety, acyl-PAF formed a significantly higher quantity of hydrogen bonds than alkyl-PAF (Fig. 4, *A* and *B*). Despite reducing van der Waals interactions, the enhanced hydrogen bonding network contributed to a stronger overall binding affinity (Fig. 4*G*). MSM analysis showed a 4.67-fold increase in the association rate (Kon) and a 0.21-fold lower dissociation rate (Koff), signifying faster binding onset and enhanced kinetic stability (Fig. 5*D*). Several synthetic and natural small compounds that target PAFR have been documented to exhibit high-affinity binding without inducing receptor activation (15, 36), highlighting that high affinity does not inherently result in effective signaling. Similarly, WEB2086 (Apafant) could inhibit PAFR signaling through enhanced hydrogen bonding and electrostatic interactions (Fig. S4).

In this study, we performed the MD simulation using the C-terminally truncated (310–342) PAFR structure, consistent with the crystal structure (PDB: 8XYD) (25). This region is predicted to be intrinsically disordered with very low confidence scores, and our preliminary simulations incorporating this region led to severe instability and poor convergence (Fig. S5). Moreover, our systems were constructed without G protein complexes. Although we observed ligand-induced ICD conformational differences relevant to G protein binding, our simulations did not explicitly include G proteins and thus could not fully address the dynamic mechanisms of recruitment and selective coupling.

**Figure 6:**
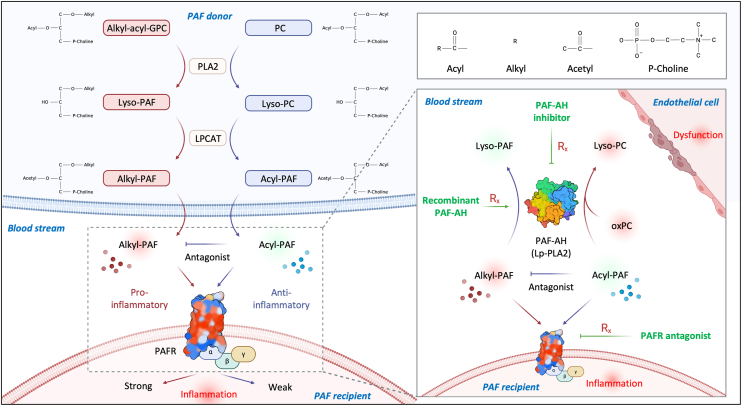
**Mechanistic insights into PAF signaling involving alkyl-PAF and acyl-PAF, from molecular dynamics to clinical relevance**. Figure created using BioRender. Alkyl-acyl-GPC, 1-alkyl-2-acyl-*sn*-glycero-3-phosphorylcholine; LPCAT, lysophosphatidylcholine acyltransferase; Lp-PLA_2_, lipoprotein-associated phospholipase A_2_; Lyso-PC, lysophosphatidylcholine; oxPC, oxidized phosphatidylcholine; PAF, platelet-activating factor; PAFR, platelet-activating factor receptor; PAF-AH, platelet-activating factor acetylhydrolase; P-Choline, phosphatidylcholine; PLA_2_, phospholipase A_2_.

Alkyl-PAF and acyl-PAF originate from distinct phospholipid precursors; however, they are produced *via* an identical enzymatic pathway intracellularly, including the release of the *sn*-2 acyl group mediated by phospholipase A_2_ (PLA_2_) and subsequent re-acylation by the lysophosphatidylcholine acyltransferase (LPCAT) (15, 37). Alkyl-PAF acts as a potent PAFR agonist, driving pro-inflammatory signaling, whereas acyl-PAF functions as a partial antagonist that may aid in the modulation of excessive inflammation (Fig. 6, left panel) (20). In human endothelial cells, the production of acyl-PAF is approximately 11-fold higher than that of alkyl-PAF (Table S1), implying a potential endogenous mechanism to counteract PAF-mediated activation (38). Following receptor binding, both analogs undergo hydrolysis by plasma PAF acetylhydrolase (PAF-AH), also known as lipoprotein-associated phospholipase A_2_ (Lp-PLA_2_) (39, 40). Nonetheless, their subsequent metabolic pathways and physiological outcomes are markedly divergent. Alkyl-PAF is converted into biologically inactive lyso-platelet-activating factor (lyso-PAF) by PAF-AH. In contrast, acyl-PAF can be hydrolyzed to produce lysophosphatidylcholine (lyso-PC) (20), another bioactive lipid associated with vascular inflammation, endothelial dysfunction, and metabolic disorders (41). Besides, PAF-AH also hydrolyzes short-chain or *sn*-2 oxidized phosphatidylcholine (oxPC) on lipoproteins, leading to the production of lyso-PC (42, 43, 44). This dual role positions PAF-AH at the center of a therapeutic paradox: it deactivates inflammatory triggers (alkyl-PAF and oxPC) while simultaneously producing pathogenic lipid mediators, the lyso-PC (Fig. 6, right panel) (45).

It is now well recognized that lipid metabolism is intricate at the intersection of signal transduction and pathological progression; therapeutic strategies that overlook this duality often fail to achieve clinical efficacy. Epidemiological data have shown that elevated PAF-AH levels correlate with increasing cardiovascular risk (46, 47, 48, 49). PAF-AH exhibits greater hydrolytic activity toward acyl-PAF than alkyl-PAF, suggesting a preferential role in generating pro-inflammatory lipid metabolites (22). Higher levels of lyso-PC or lyso-PC-enriched modified LDL, such as electronegative LDL (LDL(−)) and oxidized LDL (oxLDL), may contribute to the pathogenesis of vascular inflammation and atherosclerosis, leading to cardiometabolic disorders (16, 41). Darapladib, a selective PAF-AH inhibitor, reduced lyso-PC levels and plaque inflammation in preclinical models but failed to improve cardiovascular outcomes in phase III trials (50, 51, 52). One of the reasons could be insufficient clearance of alkyl-PAF (Fig. 6, right panel). Similarly, recombinant PAF-AH (rPAF-AH; Pafase) is designed to degrade active PAF molecules in the blood and reduce PAF-mediated cell activation and inflammatory responses, but did not show a promising effect in the phase IIb trial (53). One of the reasons could be its ability to hydrolyze acyl-PAF and oxPC, leading to the generation of pro-inflammatory lyso-PC (Fig. 6, right panel). On the other hand, PAFR antagonists demonstrated limited clinical efficacy (20, 54). PAFR blockade does not eliminate the accumulation of lyso-PC and its downstream metabolites, which may continue to fuel inflammation through PAFR-independent mechanisms (Fig. 6, right panel). These failures underscore a key challenge: Existing strategies may oversimplify a structurally intricate pathway and functionally ambivalent. Our findings offer critical insight into this dilemma. The capacity of acyl-PAF to bind PAFR without activating it reveals a built-in regulatory mechanism that dampens receptor activity. This insight highlights the limitations of conventional strategies that broadly inhibit PAFR or PAF-AH. Instead, future therapeutic strategies should adopt precision-targeted interventions that independently modulate alkyl-PAF, acyl-PAF, and lyso-PC, enabling more effective and selective treatment of PAF-driven pathologies.

## Conclusion

In summary, our study provides a structural and dynamic explanation for the partial antagonistic behavior of acyl-PAF, highlighting its ability to bind PAFR without inducing full receptor activation (Fig. S6). This ligand-specific structural modulation alters PAFR dynamics and may represent a natural checkpoint in the regulation of inflammatory signaling. By dissecting the biosynthetic, metabolic, and functional distinctions between alkyl-PAF, acyl-PAF, and lyso-PC, we underscore the need to reconsider current therapeutic strategies that target the PAF pathway as a whole. Precision approaches that selectively modulate these lipids may ultimately yield more effective and promising interventions for inflammation-driven diseases.

## Experimental procedures

### System preparation

The structure of the PAFR was retrieved from the Protein Data Bank (PDB ID: 8XYD, 2.9 Å), produced using state-of-the-art cryogenic electron microscopy (cryo-EM) (25). The G protein complex was removed, while chain A was retained for further modeling investigations. Hydrogen atoms and protonation states have been designated at physiological pH (7.4) using CHARMM-GUI (Lehigh University, Bethlehem; https://www.charmm-gui.org, assessed on May 28, 2025). The three-dimensional (3D) structures of the alkyl-platelet-activating factor C-16 (alkyl-PAF C-16; 1-hexadecyl-2-acetyl-*sn*-glycero-3-phosphorylcholine), acyl-platelet-activating factor C-16 (acyl-PAF C-16; 1-palmitoyl-2-acetyl-*sn*-glycero-3- phosphorylcholine), and WEB2086 (4-[3-[4[(2-Chlorophenyl)-9-methyl-6*H*-thieno[3,2-f][1,2,4]triazolo[4,3-a]diazepin-2-yl]-1-oxopropyl]morpholine) were constructed using the CHARMM-GUI Ligand Reader & Modeler (55). The CHARMM36 force field was chosen for the preparation and all ensuing simulations of the protein and ligands. All structural figures were visualized using PyMOL software (version 3.1, Schrödinger, LLC, USA).

### Computational molecular docking

Protein-ligand molecular docking was performed using AutoDock Vina software (version 1.1.2) to forecast the binding conformations of alkyl-PAF, acyl-PAF, and WEB2086 at the orthosteric region of PAFR (56). The PDB files of both the protein and ligands were converted to PDBQT format using Open Babel (version 3.1.1, https://openbabel.org, assessed on May 28, 2025) (57). The docking grid was centered on the orthosteric binding pocket, with a grid box size of 18 × 18 × 18 Å^3^. Docking was conducted with an exhaustive level of 64, generating 30 binding modes per ligand. Binding affinity was assessed using the Vina scoring tool, and the highest-ranked posture was chosen for further research based on an energy range threshold of 2.5 kcal/mol.

### Molecular dynamics simulation

The PAFR-membrane system was constructed using the CHARMM-GUI Membrane Builder (55), embedding the PAFR into a 75 × 75 Å POPC (1-palmitoyl-2-oleoyl-*sn*-glycero-3-phosphorylcholine) bilayer. A 15 Å thick layer of TIP3P (transferable intermolecular potential with three points) water was applied to both sides of the membrane, and 0.15 M NaCl was introduced to neutralize the system and mimic physiological ionic strength. The docked poses of alkyl-PAF and acyl-PAF were converted to GROMACS-compatible parameters using the CGenFF (CHARMM General Force Field) (58), which were subsequently integrated with the protein topology to form a unified system. All simulations were conducted using GROMACS (GROningen MAchine for Chemical Simulations, version 2024.4, https://www.gromacs.org assessed on May 28, 2025), employing the CHARMM36 force field for the protein-lipid environment and CGenFF parameters for the ligands. Energy minimization was carried out using the steepest descent algorithm until the greatest force decreased to 1000 kJ mol^-1^ nm^-1^. Equilibration proceeded in two phases: an NVT ensemble at 310 K with a velocity-rescaling thermostat, succeeded by an NPT ensemble at 1 bar using the Berendsen barostat. During these equilibration phases, positional restraints on the PAFR backbone, side chains, dihedral angles, and lipid molecules were gradually released. Production MD simulations were conducted for 1000 nanoseconds (ns) with a 2-fs (fs) time step. The system was maintained at 310 K with the velocity-rescaling thermostat, while the pressure was controlled at 1 bar using the Parrinello-Rahman barostat. Long-range electrostatic interactions were calculated using the Particle Mesh Ewald (PME) method, with van der Waals (vdW) and electrostatic cutoffs both set to 12 Å. Trajectory snapshots were recorded at 40 picoseconds (ps) intervals for further analysis of protein-ligand interactions and system stability.

### Trajectory and dynamics analysis

The stability of PAFR was evaluated using root-mean-square deviation (RMSD) and radius of gyration (Rg). Root-mean-square fluctuation (RMSF) analysis was performed to evaluate residue-level flexibility, while solvent-accessible surface area (SASA) was calculated between the PAF ligands and key PAFR residues (W73, F97, F174, E175, W255, and H275) to investigate receptor-ligand interface dynamics. Hydrogen bond and interaction energies (coulombic short-range interactions, Coul-SR; Lennard-Jones short-range interactions, LJ-SR) between the PAF ligands and PAFR were computed to assess binding affinity and hydrophobic interactions. In addition, the distance between the P162 in the extracellular loop 2 (ECL2) and the H268 (ECL3) was monitored to examine changes in the pocket entrance during ligand binding. All statistical analyses and figures were generated using Python (version 3.13, https://www.python.org, assessed on May 28, 2025) to process the GROMACS trajectory files. The time-averaged ligand occupancy map was generated using MDAnalysis. The resulting OpenDX maps (.dx format) represent the spatial probability distribution of ligand atoms across the simulation.

### Umbrella sampling simulation

An umbrella sampling simulation was performed using GROMACS (version 2024.4) to investigate the binding affinity between the PAF ligands and the PAFR. TIP3P water and sodium chloride ions were included for solvation and ionization, respectively. The system underwent energy minimization and was then equilibrated under NPT conditions (310 K, 1 bar) to remove steric clashes and achieve a stable initial configuration. A 1500 ps steered pulling simulation was conducted at a pulling rate of 0.01 nm/ps with a spring constant of 1000 kJ mol^-1^ nm^-2^ along the z-axis. The position restraints on residues within 12 Å of the PAF binding pocket were removed during the simulation to ensure sufficient flexibility. Umbrella windows were selected from 0 to 5 nm based on the center-of-mass (COM) distance, and each window was simulated for 2 ns using a velocity-rescaling thermostat (310 K) and a Parrinello–Rahman barostat (1 bar). Long-range electrostatic interactions were calculated using the PME approach, with vdW and electrostatic cutoffs set to 14 Å. The potential of mean force (PMF) was computed using the Weighted Histogram Analysis Method (WHAM) modules with bootstrapping. Finally, the absolute binding free energy (Δ*G*°) was determined by applying the standard-state correction, as shown in Equations (1, 2).

(1) $$\Delta G{}^{\circ}={-\Delta G}_{PMF}+{\Delta G}_{v}$$

(2) $${\Delta G}_{v}=-RT\;\ln\left(\frac{V_{sim}}{V^{o}}\right)$$

In Equation (1), Δ*G*_*PMF*_ represents the free energy differential associated with the PMF (bound vs unbound), while Δ*G*_*V*_ denotes the volume correction defined in Equation (2). Δ*G*° denotes the absolute binding free energy under the standard state (1 M), *R* is the gas constant, *T* is the temperature, *V*_*sim*_ represents the simulation box volume, and *V*^*o*^ is the standard-state volume corresponding to 1 M concentration.

### Markov state models

A MSM was constructed from the molecular dynamics trajectories to elucidate the binding and unbinding kinetics between the PAF ligands and the PAFR. The PAF-PAFR distance distribution and free energy landscape were computed to identify the bound and unbound states. The cutoff distances were determined by locating inflection points in the distance profiles through second-derivative analysis, corresponding to 1.293 nm for alkyl-PAF and 1.384 nm for acyl-PAF, respectively. All analyses relating to MSM were conducted using PyEMMA (version 2.5.12) (59). To ensure Markovian behavior, an implied timescale analysis was performed for various lag times (τ), and the Chapman-Kolmogorov test was used to validate the consistency of state-to-state transitions. Each trajectory frame was assigned to the bound or unbound state according to the chosen distance cutoff, resulting in a two-state model. The means binding duration (average residence time in the bound state), means unbinding duration (average residence time in the unbound state), and the switch count (number of transitions between bound and unbound) were calculated from these state assignments. We subsequently constructed a transition probability matrix and computed the association (Kon) and dissociation (Koff) rate constants.

## Data availability

All data generated or analyzed during this study are included in this published article and its supporting information files.

## Supporting information

This article contains supporting information (60, 61, 62, 63, 64, 65).

## Conflict of interest

The authors declare that they have no conflicts of interest with the contents of this article.
