# Supplementary material for: Structural and molecular dynamics insights into the competitive inhibition of the platelet-activating factor receptor by acyl-PAF
Source: J Biol Chem. 2025 Oct 16;301(12):110831. doi: 10.1016/j.jbc.2025.110831 (PMC12648956; doi:10.1016/j.jbc.2025.110831)
Supplement: Supplementary File 2 [file mmc2.docx]

**Supporting information**

**Structural and molecular dynamics insights into the competitive inhibition of the platelet-activating factor receptor by acyl-PAF**

Shao-Chi Hung, Chih-Chieh Chen, Hua-Chen Chan, Mei-Lin Chan, You Shan Ngui, Jia-Bin Mei, Yi-Wen Huang, Vyala Hanumanthareddy Chaithra, Gopal Kedihithlu Marathe, Liang-Yin Ke

**Table of contents**

Figure S1 (Page. S-1)

Figure S2 (Page. S-2)

Figure S3 (Page. S-3)

Figure S4 (Page. S-4)

Figure S5 (Page. S-5)

Figure S6 (Page. S-6)

Table S1 (Page. S-6)

**Page. S-1**


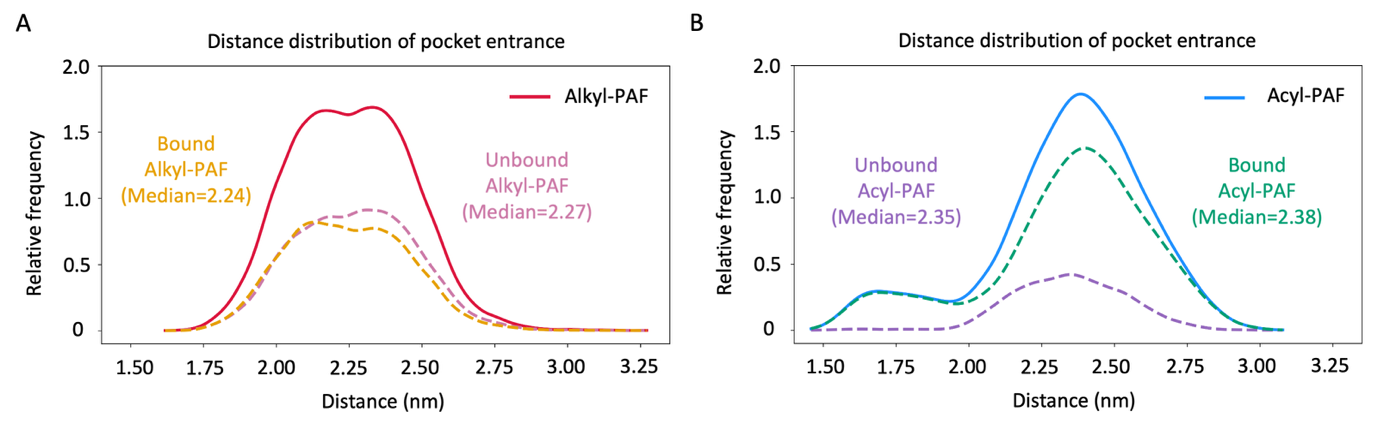


**Figure S1.** Distance distribution of pocket entrance in alkyl-PAF and acyl-PAF systems. The thresholds distinguishing bound and unbound states were determined using the second-derivative analysis of the free energy landscape (**Fig. 5B**), with cutoff values of 1.293 nm for alkyl-PAF and 1.384 nm for acyl-PAF. (A) In the alkyl-PAF-PAFR complex, the bound state exhibits a smaller pocket opening (median = 2.24 nm; yellow) compared with the unbound state (median = 2.27 nm; pink). (B) In the acyl-PAF-PAFR complex, the bound state exhibits a wider pocket opening (median = 2.38 nm; green) compared with the unbound state (median = 2.35 nm; purple).

**Page. S-2**


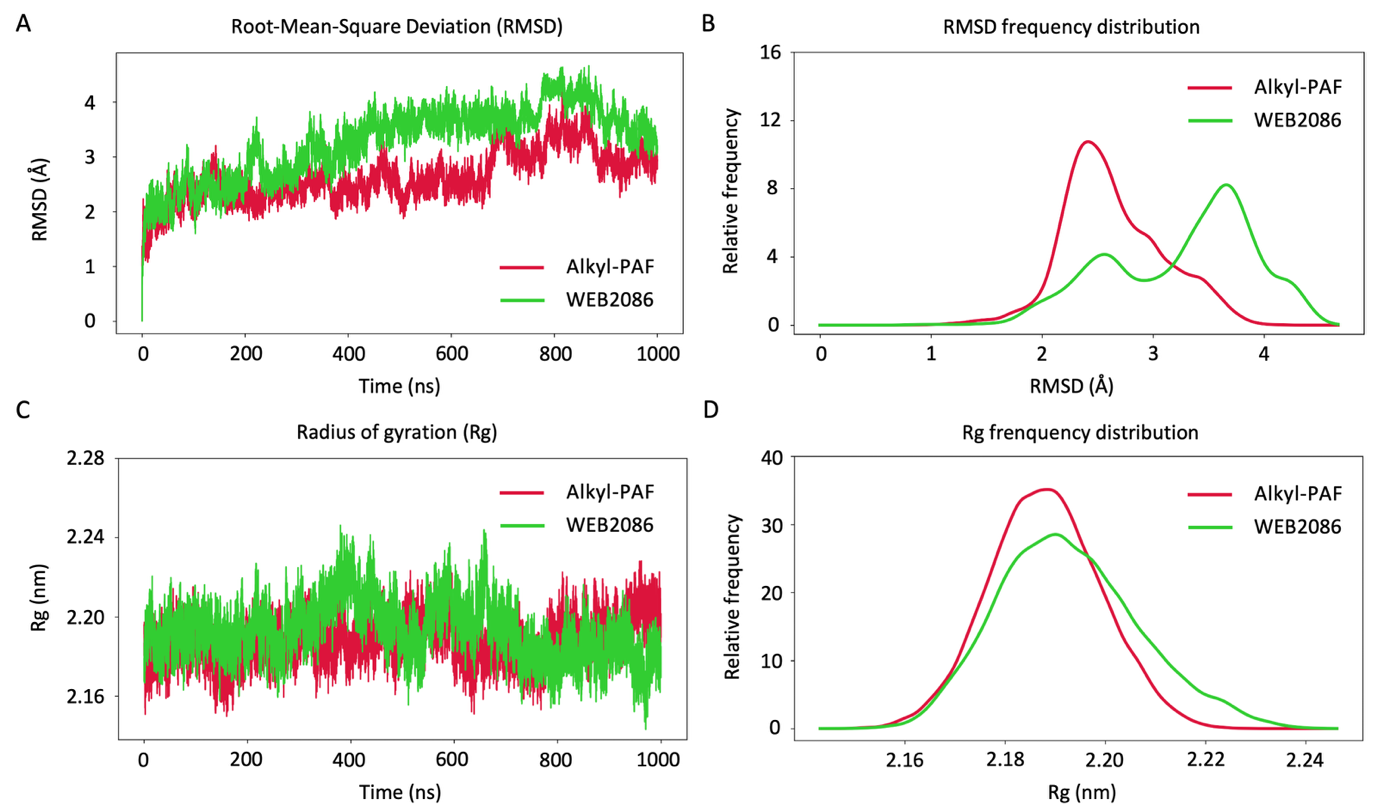


**Figure S2.** Comparative analysis of PAFR structural dynamics and in alkyl-PAF and WEB2086 systems. (A) Time evolution and (B) frequency distribution of the PAFR backbone RMSD. (C) Time evolution and (D) frequency distribution of the PAFR backbone Rg.

**Page. S-3**


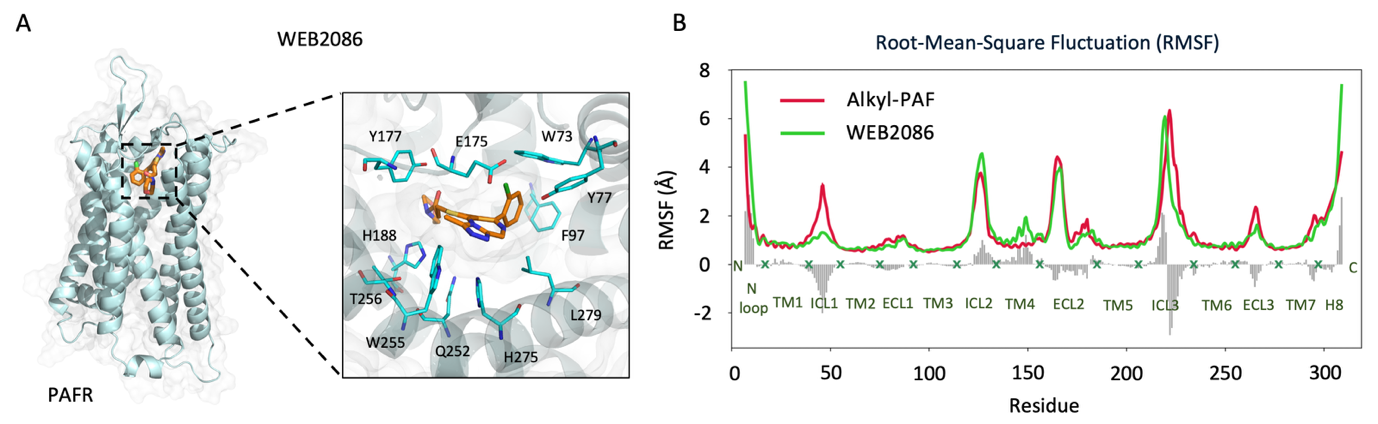


**Figure S3.** Binding characteristics and dynamic impact of WEB2086 on PAFR. (A) Docking pose of the PAFR antagonist WEB2086 within the orthosteric binding site, highlighting key interacting residues. (B) Comparative Root-mean-square fluctuation (RMSF) analysis of the PAFR backbone during molecular dynamics simulations with Alkyl-PAF (Red) and WEB2086 (Green). Gray bars represent fluctuation differences between residues in the two ligand-bound systems (WEB2086 minus alkyl-PAF); positive values indicate higher fluctuations in the WEB2086 system.

**Page. S-4**


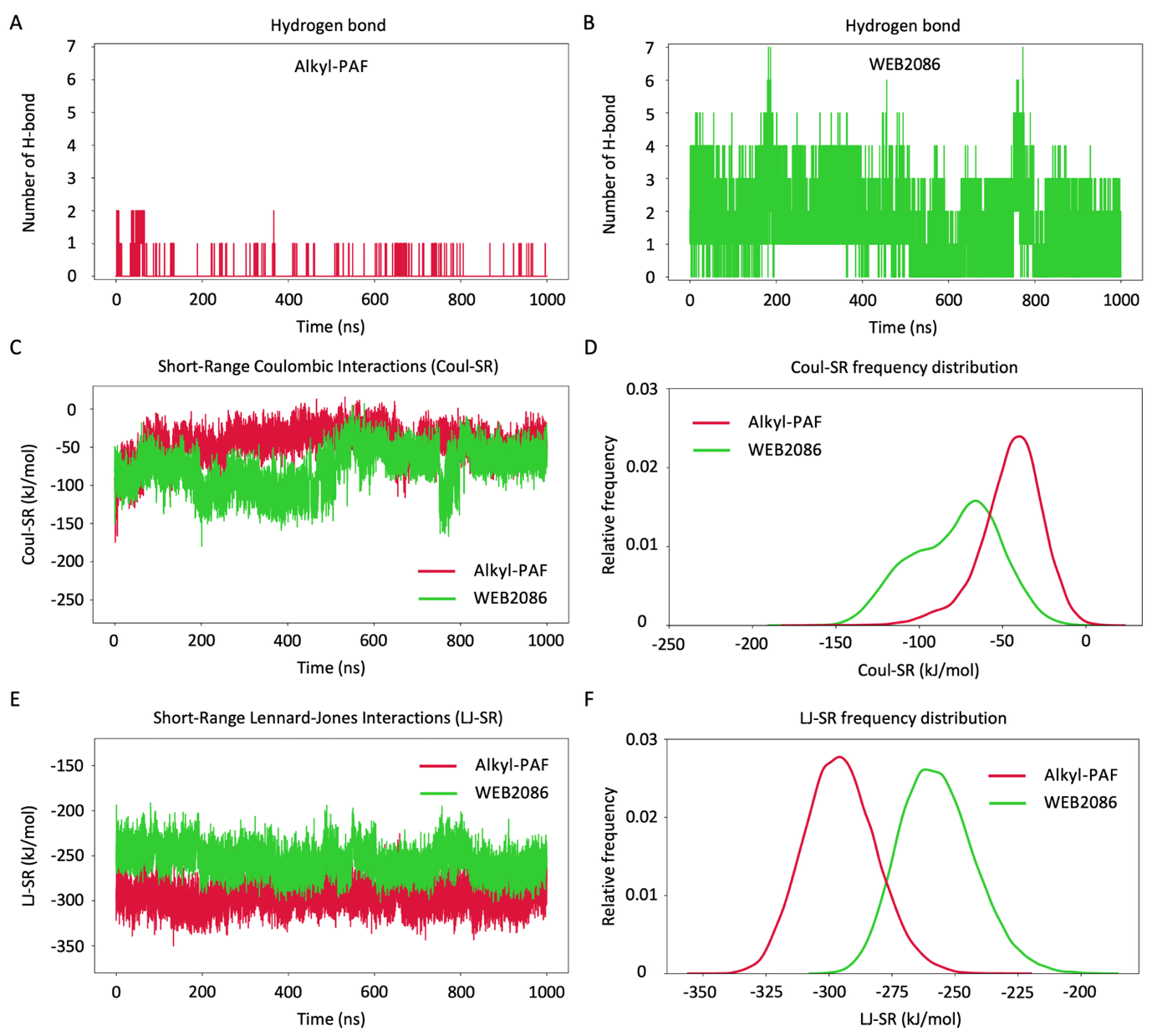


**Figure S4.** Comparative analysis of PAFR energy decomposition between alkyl-PAF and WEB2086. (A) The number of hydrogen bonds between PAFR and alkyl-PAF or (B) WEB2086. (C) Time evolution and (D) frequency distribution of the Coul-SR between PAFR and ligands (acyl-PAF and WEB2086). (E) Time evolution and (F) frequency distribution of the LJ-SR. Abbreviation: Coul-SR; short-range coulombic interactions, LJ-SR; short-range Lennard-Jones interactions.

**Page. S-5**

**
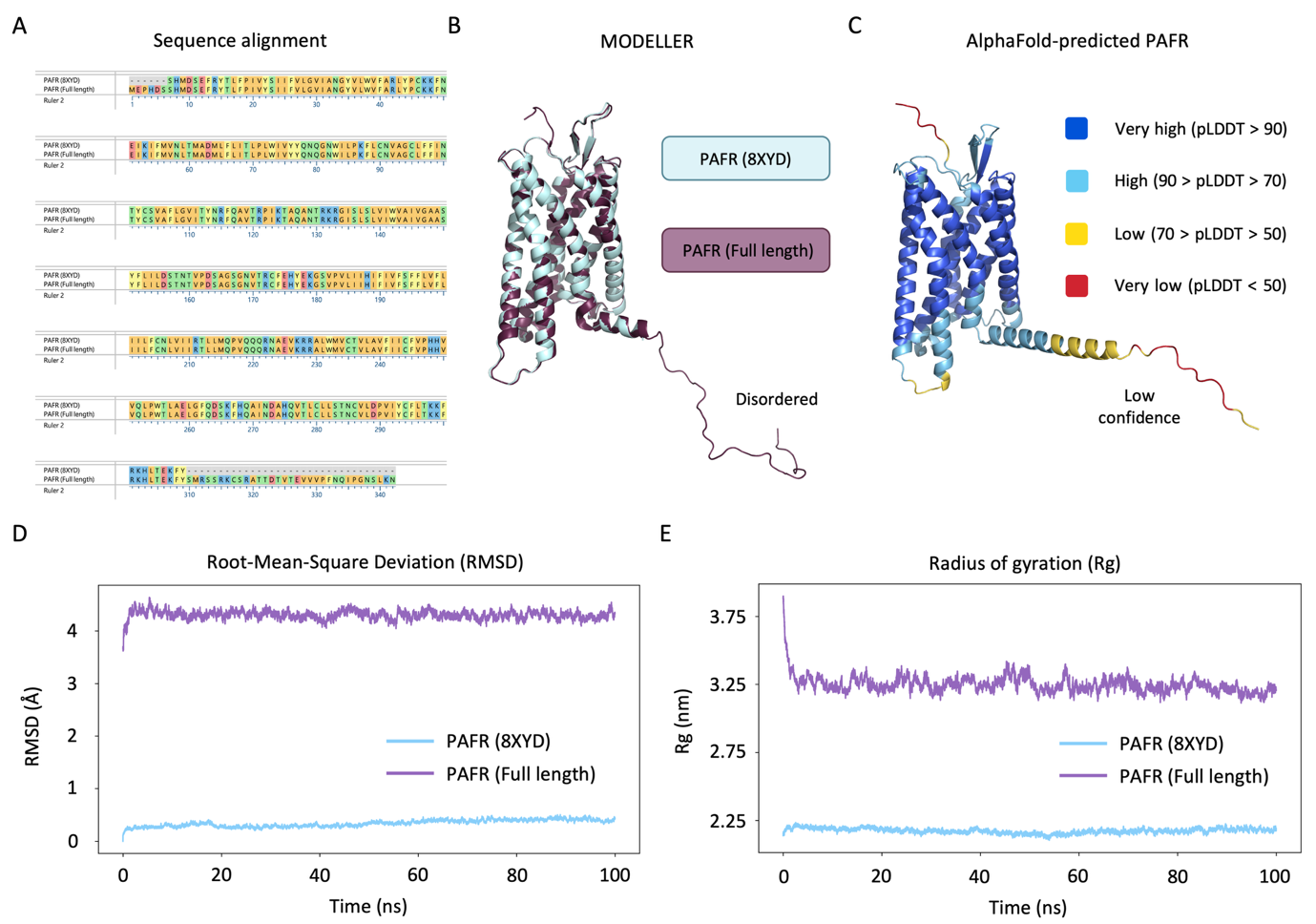
**

**Figure S5.** Comparison of full-length PAFR models reveals instability in disordered N- and C-terminal regions. (A) Sequence alignment between the cryo-EM structure of PAFR (PDB: 8XYD) and the full-length sequence shows missing regions at the N-terminus (residues 1-6) and C-terminus (residues 310-342). (B) Structural overlay of the PAFR (8XYD) (light blue) and a PAFR (full-length) built through MODELLER (purple) reveals a highly disordered C-terminal tail. (C) AlphaFold prediction of full-length PAFR with pLDDT color scoring demonstrates low confidence scores at both terminal regions. (D and E) Molecular dynamics simulations (100 ns) comparing the PAFR (8XYD) structure and the MODELLER-generated PAFR (Full-length) model show significantly higher RMSD and Rg in the PAFR (Full-length) model, indicating poor convergence and structural instability.

**Page. S-6**

**
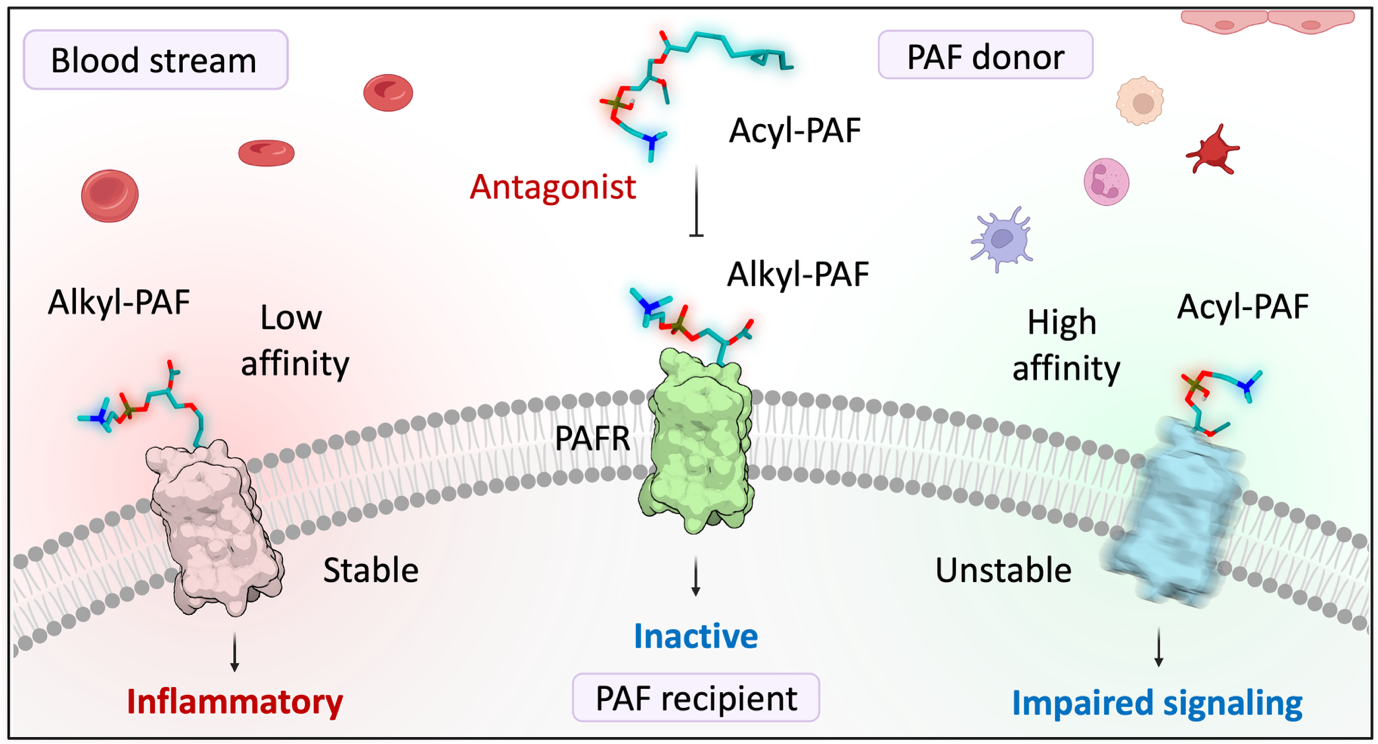
**

**Figure S6.** Graphical abstract of differential PAFR regulation by alkyl-PAF and acyl-PAF.

**Page. S-7**

**Table S1.** A literature survey of the concomitant synthesis of alkyl-PAF and acyl-PAF in various cells or tissues.

| Cell or tissue type | Source | Stimulus | Fold changes | Reference |
| --- | --- | --- | --- | --- |
| Endothelial cells | Human | A23187 | 11.37 | (1) |
|  | Bovine (Culture) |  | 2.41 |  |
|  | Bovine (*In situ*) |  | 2.30 |  |
| Bone marrow-derived  mast cells | Murine | Antigen | 3.71 | (2) |
|  |  | PMSF + antigen | 5.18 |  |
|  |  | A23187 | 2.66 |  |
|  |  | PMSF + A23187 | 3.50 |  |
| Platelets (washed) | Rabbit | A23187 | 2.62 | (3) |
| Human umbilical vein  endothelial cells | Human | No stimulus | – | (4) |
|  |  | H_2_O_2_ | 24.46 |  |
|  |  | Histamine | 17.48 |  |
| Cerebra | Bovine | No stimulus | 28.20 | (5) |
| Cerebra | Bovine | No stimulus | 18.28 | (6) |

Fold changes were calculated by using actual values of acyl-PAF over alkyl-PAF provided in the respective references.

**References**

1. Whatley, R., Clay, K., Chilton, F., Triggiani, M., Zimmerman, G., McIntyre, T., andPrescott, S. (1992) Relative amounts of 1-O-alkyl-and 1-acyl-2-acetyl-sn-glycero-3-phosphocholine in stimulated endothelial cells Prostaglandins **43**, 21-29,

2. Triggiani, M., Fonteh, A. N., andChilton, F. (1992) Factors that influence the proportions of platelet-activating factor and 1-acyl-2-acetyl-sn-glycero-3-phosphocholine synthesized by the mast cell Biochem J **286**, 497-503,

3. Chap, H., Mauco, G., Simon, M., Benveniste, J., andDouste-Blazy, L. (1981) Biosynthetic labelling of platelet activating factor from radioactive acetate by stimulated platelets Nature **289**, 312-314,

4. Weintraub, S. T., Satsangi, R. K., Sprague, E. A., Prihoda, T. J., andPinckard, R. N. (2000) Mass spectrometric analysis of platelet-activating factor after isolation by solid-phase extraction and direct derivatization with pentafluorobenzoic anhydride J Am Soc Mass Spectrom **11**, 176-181,

5. Tokumura, A., Kamiyasu, K., Takauchi, K., andTsukatani, H. (1987) Evidence for existence of various homologues and analogues of platelet activating factor in a lipid extract of bovine brain Biochem Biophys Res Commun **145**, 415-425,

6. Tokumura, A., Takauchi, K., Asai, T., Kamiyasu, K., Ogawa, T., andTsukatani, H. (1989) Novel molecular analogues of phosphatidylcholines in a lipid extract from bovine brain: 1-long-chain acyl-2-short-chain acyl-sn-glycero-3-phosphocholines J Lipid Res **30**, 219-224,
